# Radiation-induced skin regeneration: A comparative efficacy and safety analysis of alpha, beta, and gamma modalities in murine models

**DOI:** 10.14202/vetworld.2025.1168-1179

**Published:** 2025-05-17

**Authors:** Madyan Ahmed Khalaf, Marwan Noori Mohammed, Baida M. Ahmed, Sahar A. H. Al-Sharqi

**Affiliations:** 1 Department of Physics, College of Sciences, Mustansiriyah University, Baghdad, Iraq; 2 Department of Science, College of Basic Education, Mustansiriyah University, Baghdad, Iraq; 3 Department of Biology, College of Sciences, Mustansiriyah University, Baghdad, Iraq

**Keywords:** alpha radiation, beta radiation, gamma radiation, ionizing radiation, platelet-derived growth factor, skin regeneration, vascular endothelial growth factor, wound healing

## Abstract

**Background and Aim:**

The therapeutic application of ionizing radiation in wound healing, especially with alpha, beta, and gamma modalities, remains largely unexplored despite its potential for enhancing regenerative processes. This study aimed to comparatively analyze the efficacy and safety of alpha radiation (IG-A), beta radiation (IG-B), and gamma radiation (IG-G) modalities in promoting skin regeneration using a murine model of full-thickness excisional wounds.

**Materials and Methods:**

Twenty male BALB/c mice were randomized into four groups (n = 5 per group): IG-A, IG-B, IG-G, and an untreated control group (CG). Following surgical induction of full-thickness wounds (8 mm diameter), irradiation groups received 15 min of exposure at four intervals post-surgery using americium-241 (alpha), strontium-91 (beta), and cesium-137 (gamma). Wound healing was monitored macroscopically and microscopically on days 0, 2, 4, 6, 8, and 10. Histological and biochemical assessments included collagen synthesis, epithelialization, neovascularization, and growth factor (vascular endothelial growth factor [VEGF] and platelet-derived growth factor [PDGF]) quantification. Statistical analysis was performed using a one-way analysis of variance.

**Results:**

IG-A significantly accelerated wound healing, achieving approximately 100% wound closure by day 10 compared to 90% and 80% in beta and gamma radiation groups, respectively. Control wounds demonstrated only 38% closure. Histopathological analysis indicated enhanced collagen deposition, neovascularization, sebaceous gland regeneration, and complete epithelialization primarily in the alpha-treated group. Biochemical assays revealed significantly elevated VEGF and PDGF levels in irradiated groups, with IG-A exhibiting the highest expression.

**Conclusion:**

IG-A demonstrated superior efficacy in accelerating wound healing and tissue regeneration compared to beta and gamma modalities. This novel finding suggests a potential therapeutic role for IG-A in clinical wound management strategies.

## INTRODUCTION

The skin, accounting for approximately 15% of the total body weight in adults [1], functions as the primary protective organ [2], offering essential defense against external threats [3]. The integumentary system, which includes the epidermis, dermis, and subcuta-neous tissue, significantly contributes to this protective role [4, 5]. A wound represents a disruption in the continuity of the epithelial barrier, potentially impairing both tissue structure and physiological functions [6, 7]. Wounds can result from various factors, including trauma [8], burns [9], lacerations, or abrasions [10]. The wound-healing process is highly intricate [11] and involves four interconnected stages: Hemo-stasis, inflammation, proliferation, and remodeling of conn-ective tissue [12]. These phases work synergistically to respond effectively to injury and reestablish a fully functional epidermal barrier [13]. As such, wound healing is critical in evaluating the effectiveness of healthcare interventions [14]. Consequently, exploring methods to expedite wound healing is of considerable importance [15].

In addition to biological factors influencing healing, biophysical treatments such as low-energy laser therapy [16], ultraviolet light [17], pulsed radiofrequency radiation [18], and pulsed electromagnetic fields [19] have been identified as potential accelerators of wound repair [20]. Radiation involves energy release from specific sources, whether natural or artificial [21]. Various radiation forms exist, including high-energy particles capable of inducing ionization either directly (e.g., alpha and beta particles) or indirectly (e.g., gamma rays) [22]. Typically, these radiation types are classified into charged particles and electromagnetic waves [23].

Although radiotherapy is widely recognized for cancer treatment, its potential application in non-cancerous conditions such as wound healing remains significantly underexplored. Studies by Omer [24] and Cuttler [25] address this research gap by aiming to evaluate the therapeutic effects of low-dose ionizing radiation (LDI) on skin regeneration. While radiotherapy effectively manages various cancer types, serving purposes ranging from curative to palliative care [26], treatment planning involves careful consideration of biological and technical factors to optimize outco-mes [27]. Previous research has examined low-dose irradiation in wound healing. For instance, Susanto *et al*. [28] found that radiation sterilization using radioactive cobalt combined with freeze-drying effec-tively preserved bone transplants, showing promising clinical applications [29]. Conversely, Maria-De-Almeida *et al*. [30] demonstrated that electron irradiation impeded the wound repair process in rat models. Song *et al*. [31] proposed that low-dose X-ray irrad-iation (≤1 Gy) promotes fracture healing by enhancing vascular endothelial growth factor (VEGF) expression. Lilge *et al*. [32] evaluated the feasibility of low-level laser therapy by transilluminating wound dressings, concluding that further studies are necessary to confirm therapeutic effectiveness. In addition, Zhang *et al*. [33] observed accelerated wound healing in male rats exposed to low-dose X-ray irradiation.

However, despite these individual studies, a systematic comparative analysis examining the efficacy and safety of charged-particle radiation (alpha and beta) versus electromagnetic radiation (gamma) for full-thickness excision wounds is missing from the curr-ent literature. Furthermore, existing studies have not sufficiently clarified the distinct mechanisms by which different radiation types may influence critical processes such as growth factor expression, collagen synthesis, re-epithelialization, and neovascularization, which are essential for effective wound healing. Previous stu-dies have primarily emphasized high-dose radiation’s detrimental effects, leaving a gap in understanding how controlled low-dose radiation could be beneficial therapeutically without adverse side effects.

This study seeks to bridge this critical gap by comparatively analyzing the effects of alpha, beta, and gamma radiation on wound healing in murine models, employing macroscopic, histological, and biochemical assessments. Through this approach, we aim to provide new insights into the regenerative potential of ionizing radiation, potentially leading to innovative, non-invasive clinical strategies for enhanced wound management and tissue repair.

**Figure 1:**
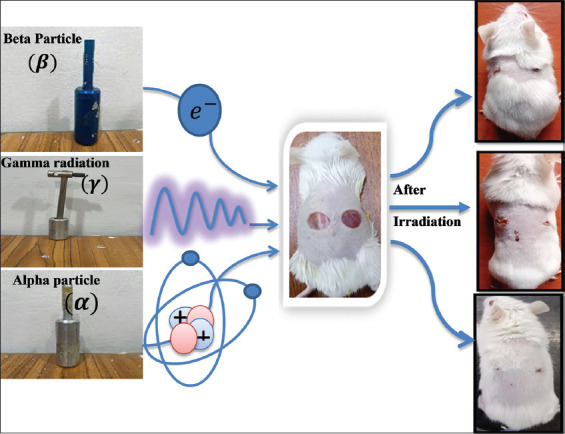
Representative images of treating mice wounds with alpha, beta, and gamma rays for 15 min.

Building on prior research highlighting the adverse effects of particle radiation (electrons) and the beneficial effects of photon radiation (X-rays and gamma rays), this investigation specifically explores low-dose ionizing radiation’s impact on wound healing. The systemic effects of photon radiation (gamma rays) and particle beams (alpha radiation [IG-A] and beta radiation [IG-B]) on wound healing in male mice were investigated. This study evaluates both direct (alpha and beta rays) and indirect (gamma rays) radiation effects as illustrated in Figure 1 using comprehensive histo-logical and biochemical analyses alongside macroscopic assessments.

## MATERIALS AND METHODS

### Ethics approval

All procedures involving animals were strictly followed guidelines set by the National Research Council (2011) and were approved by the Institutional Animal Care and Use Committee of the College of Science, Mustan-siriyah University, Baghdad, Iraq (reference number: BCSMU/1123/0001Ph).

### Study period and location

The study was conducted from January 11, 2024, to January 21, 2024, at the animal house of the College of Science, Mustansiriyah University, Baghdad, Iraq.

### Mice

The albino mouse model was developed in our research facility using male BALB/c mice, obtained from the Iraqi Center for Genetics and Cancer Research at Mustansiriyah University in Baghdad, Iraq. These mice were aged between 10 and 12 weeks at the commencement of the study. On arrival, all mice were provided at least 72 h to acclimate to their new environment. An innovative experimental design was adopted, systematically dividing the mice into four distinct groups, each comprising five individuals, to evaluate the regenerative effects of alpha, beta, and gamma radiation within a controlled framework. Mice were maintained in cages at an animal facility accredited by the Association for Assessment and Accreditation of Laboratory Animal Care. Environmental conditions were consistently maintained with a 12-h light-dark cycle, a temperature of 26°C ± 2°C, and a relative humidity of 50% ± 20%. Mice received water and adequate commercial rodent feed sourced from the Iraq Feed Company (Erbil, Iraq).

### Skin wounding and experimental grouping

The injury protocol consisted of two steps: Shaving the dorsal skin and creating the wound, both performed under anesthesia using 80 mg/kg of 10% ketamine combined with 10 mg/kg xylazine. Initially, hair on the dorsal area was shaved using an electric clipper. Subsequently, a circular wound of approximately 100 mm² was created on the dorsal skin using a sterile steel punch (Kai Medical, Chiyoda, Japan) disinfected with 70% ethanol. This standardized procedure was uniformly applied to all mice across the experimental groups. After injury induction, the mice were housed in sanitary cages containing autoclaved bedding, with non-lethal wounds left uncovered [34]. Twenty healthy young albino mice were randomly assigned to four groups. Each mouse was individually identified by marking its tail or body, or left unmarked, before initiating the study.

1. Group I (control group [CG]): Received no treatment.
2. Group II (IG-A): Exposed to IG-A for 15 min, repeated 4 times.
3. Group III (IG-B): Exposed to IG-B for 15 min, repe-ated 4 times.
4. Group IV (IG-G): Exposed to gamma radiation for 15 min, repeated 4 times.

### Experimental design, treatment, and irradiation protocol

**Figure 2:**
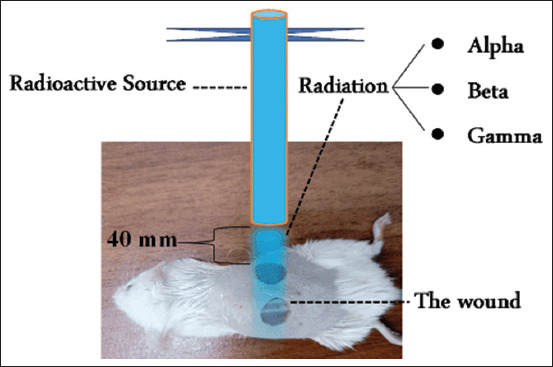
Schematic of the experimental methodology.

Three distinct radioactive sources were emp-loyed for irradiation: americium-241, strontium-91, and cesium-137, as detailed in Table 1. The mice were immobilized using a specialized apparatus that allowed normal breathing but restricted bodily movements. Each mouse was placed inside an enclosure, and its anterior (non-exposed) skin region was shielded entirely with aluminum foil to prevent exposure to alpha and IG-B. The radioactive source was positioned 40 mm from the two dorsal wounds, as illustrated in Figure 2. Radiation was administered 4 times, on alternating days, with each exposure lasting 15 min. Wounds were closely monitored and documented at regular intervals: Day 0, day 2, day 4, day 6, day 8, and day 10 following surgery. Adjacent to each wound, a measurement device was placed, and digital photographs were captured perpendicularly to the wound surface for documentation purposes.

**Table 1:** The significant effect of irradiation on the wound-healing process in albino mice, as evidenced by the statistically significant differences in the treated groups compared with the CG (p < 0.05).

Groups	Day 0	Day 2	Day 4	Day 6	Day 8	Day 10
CG	99.98 ± 2.13	89.95 ± 3.96	83.56 ± 4.03	78.70 ± 3.40	77.61 ± 3.37	60.87 ± 2.74
IG-G	99.02 ± 1.65	84.43 ± 4.41	74.82 ± 4.09	56.49 ± 3.44	34.34 ± 2.96	19.82 ± 1.93
IG-B	101.55 ± 1.79	79.20 ± 3.34	71.74 ± 2.39	49.37 ± 2.96	17.84 ± 1.38	9.57 ± 1.26
IG-A	101.82 ± 1.55	71.20 ± 2.36	28.54 ± 2.39	18.48 ± 1.04	6.31 ± 1.01	Healed

CG=Control group, IG-A=Alpha radiation, IG-B=Beta radiation, IG-G=Gamma radiation

### Blood collection and euthanasia

On the tenth day, blood samples were collected from anesthetized mice through cardiac puncture using a microsyringe. The collected blood was placed into tubes containing Gel and Clot Activator. After allowing coagulation for at least 30 min at 26°C, serum was separated by centrifuging the samples at 3000 × *g* for 10 min and immediately stored at −80°C. Subsequently, whole blood samples (minimum of 0.4 mL per sample) were examined using a hematology analyzer (Element HT5, Heska, Loveland, CO, USA) according to the manufacturer’s instructions. Following blood collection, the mice were euthanized by cervical dislocation, and skin samples were collected for subsequent histological analysis.

### Histological analysis

Regenerated tissue samples from all four experimental groups were collected on day 10 and prepared for histological examination. The collected samples were fixed using 10% buffered formalin solution and subsequently embedded in paraffin at temperatures ranging between 40°C and 60°C. The paraffin-embedded tissues were then sectioned into 6 μm-thick slices using a microtome. To identify possible histological changes, the tissue sections were stained with hematoxylin and eosin, followed by microscopic examination [35]. The results obtained from the irrad-iated groups were analyzed and compared with those from the CG.

### Measurement of wound contraction, reduction, and epithelialization

The reduction in wound size was evaluated using a ruler to measure the wound diameter. The wound area was calculated using planimetric measurements performed every other day until healing was complete. Wound contraction was determined using a specific formula [36].


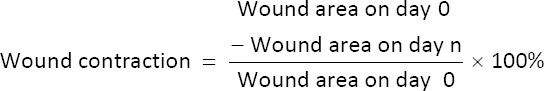


Where n = Days on which the measurement was performed.

Epithelialization time was determined by counting the number of days taken for dead tissue remnants to completely fall off, leaving no raw wound behind. This was calculated using a specific formula [37].

Wound reduction = Wound area on day 0−Wound area on day n

Wound epithelialization = Wound area of reduction − Wound area of contraction

### Statistical analysis

Quantitative data were analyzed using Microsoft Excel 2019 (Microsoft Corp., Redmond, WA, USA) and ImageJ (National Institutes of Health, Bethesda, Maryland, USA) software. One-way analysis of vari-ance was employed to assess differences among the experimental groups. All results are expressed as mean ± standard deviation (SD). p < 0.05 was considered statistically significant.

## RESULTS

### Macroscopic evaluation of wounds

Following the surgical procedure, all mice remained in good health and showed no signs of infection. Microscopic analysis confirmed that a sterile environment was consistently maintained across all experimental groups during the injury process. To assess the effects of contraction and re-epithelialization, histological comparisons were made between the irrad-iated groups and the CG.

On day 0, there were no significant differences between the treatment groups and the CG. By day 2, the CG wounds began showing signs of inflammation, with a reduction in area observed in one of the two wounds. In contrast, IG-G exhibited a noticeable reduction in wound area with minimal inflammation compared to the CG. Similarly, IG-A and IG-B showed marked decreases in wound size. By day 4, delayed healing and infection were observed in the CG, whereas the irradiated groups – especially IG-A – demonstrated accelerated wound closure. These findings highlight a previously unreported role of IG-A in mitigating wound-related complications.

**Figure 3:**
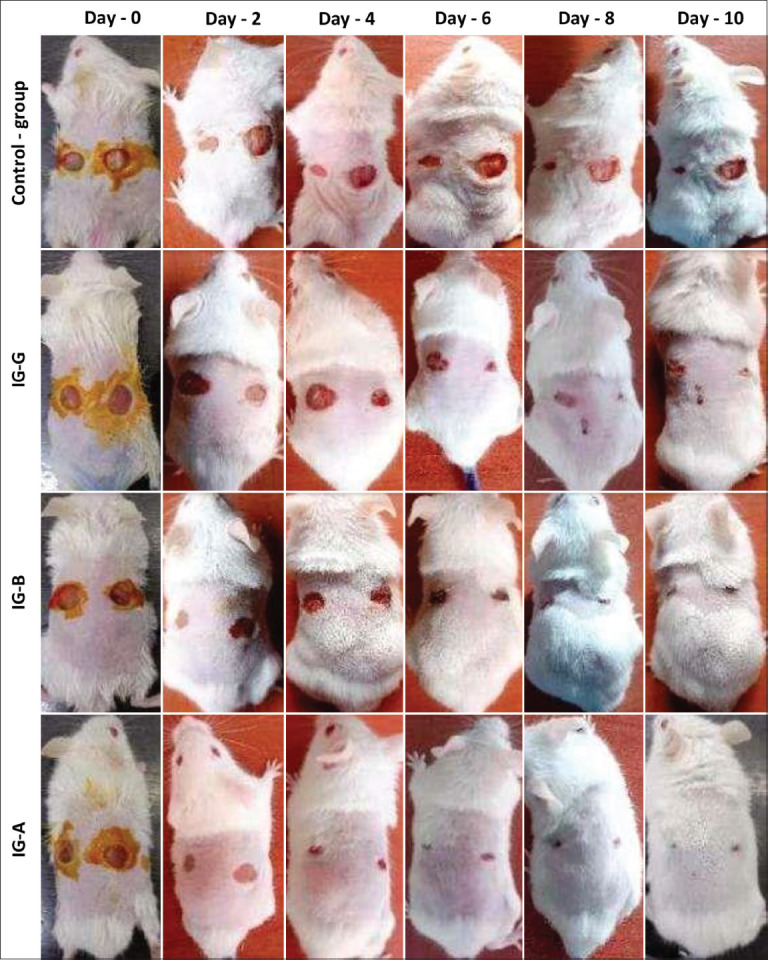
Photographs showing wound monitoring of full-thickness skin excision in albino mice at different post-lesion time points. The sampling is for re-epithelialization analysis.

IG-B also showed reduced inflammation and improved wound appearance relative to the gamma-irradiated group. IG-A displayed the most pronounced reduction in wound area. In the CG, inflammation persi-sted until approximately day 9. In contrast, IG-A achieved complete wound healing by day 10 (approximately 100%). All irradiated groups (alpha, beta, and gamma) exhibited significantly greater reductions in wound area compared to the CG, as illustrated in the sequence of digital images in Figure 3.

Wound healing progression in the mice was recorded and analyzed over time, with detailed data presented in Table 1. Wound contraction, reduction, and epithelialization rates were quantified by measuring wound areas at defined intervals (Figure 3). The data clearly indicate that ionizing radiation enhances the wound healing process relative to the control. On day 10, wound healing percentages were approximately 100% for IG-A, 90.58% for IG-B, 79.99% for IG-G, and 37.49% for the CG. Furthermore, significantly higher healing rates were observed in the irradiated groups on days 6 and 8 compared to the CG.

A markedly shortened epithelialization period was observed in the irradiated groups, with complete epithelialization achieved within 10 days. In contrast, the CG did not exhibit complete epithelialization within the duration of the experiment. Wound cont-raction and epithelialization both occur during the proliferation phase. Although these processes are not directly interdependent, contraction may support the progression of epithelialization by reducing wound size and the extent of extracellular matrix required for tissue regeneration, thereby promoting more rapid closure [38].

**Figure 4:**
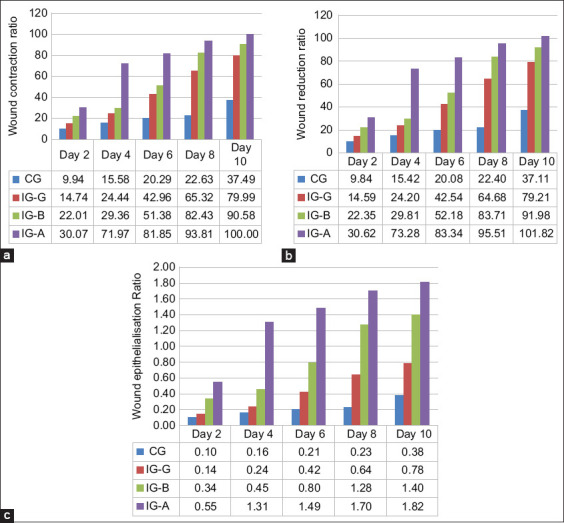
A percentage statistical analysis was conducted on the wound diameters of mice in both the treatment and control groups at the time points of days 0, 2, 4, 6, 8, and 10. (a) The analysis included evaluating the contraction healing time of the wounds in the four groups of mice, (b) comparing the ratios of wound area reduction, and (c) examining the ratios of wound area epithelialization. The data were presented as the mean ± standard deviation, with a significance level of p < 0.05.

In the group treated with IG-A, the rate of re-epithelialization reached 100% (p < 0.05). Compar-atively, beta and gamma radiation groups exhibited lower re-epithelialization rates, as illustrated in Figure 4a-c. On day 10 post-surgery, re-epithelialization percentages were approximately 100% for IG-A, 90% for IG-B, and 80% for IG-G. The CG demonstrated the slowest re-epithelialization, measured at only 40%.

### Coordinated actions of VEGF and PDGF

A study by Sadeghi-Ardebili *et al*. [39] has demonstrated that growth factors such as platelet-derived growth factor (PDGF) and VEGF play critical roles in regulating the various phases of the wound healing process. These factors are secreted by multiple cell types located near the wound site. In the present study, direct application of growth-stimulating irradiation significantly enhanced the healing rate, as reflected in the results shown in Table 2.

**Table 2:** Levels of the growth factors PDGF and VEGF in the irradiated and CG groups.

Growth factor	CG	IG-G	IG-B	IG-A
PDGF (pg/mL)	122.01 ± 4.67	192.95 ± 6.01	232.87 ± 5.40	288.12 ± 9.11
VEGF (pg/mL)	34.97 ± 3.88	66.95 ± 4.74	97.62 ± 4.23	123.04 ± 6.14

PDGF=Platelet-derived growth factor, VEGF=Vascular endothelial growth factor, CG=Control group, IG-A=Alpha radiation, IG-B=Beta radiation, IG-G=Gamma radiation

**Figure 5:**
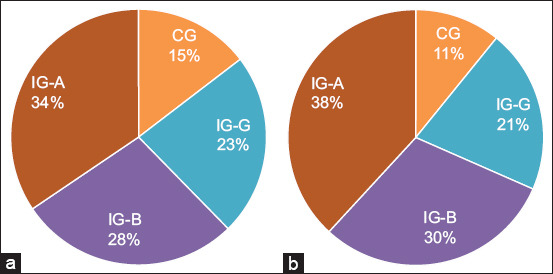
Synergistic effects of upregulated (a) platelet-derived growth factor and (b) vascular endothelial growth factor signaling on skin-wound healing in mice treated with alpha, beta, and gamma radiation, as well as the cont-rol group.

Polymerase chain reaction analysis revealed elevated expression levels of both PDGF and VEGF in the irradiated groups on day 10 when compared with the CG. Figure 5 illustrates the corresponding percentages of PDGF and VEGF detected in blood serum on day 10, indicating a statistically significant increase in the tre-ated groups (p < 0.05).

Wound healing can be adversely affected by several factors, including prolonged inflammation resulting from reduced chemotactic and phagocytic activity of neutrophils and macrophages, diminished growth factor levels, impaired collagen synthesis, and limited granulation tissue formation [40]. Platelets are widely recognized as a key source of growth factors and cytokines, making them essential contributors to tissue regeneration in the field of regenerative medicine.

### Histological analysis

Following staining with hematoxylin and eosin, wound tissues were examined microscopically to assess key histological indicators of wound healing. These included granulation tissue formation, re-epith-elialization, neovascularization, epidermal hyperplasia, presence of foreign debris, surface depression, collagen deposition, and inflammatory infiltration. Histological evaluation was conducted on day 10 to analyze these features.

**Figure 6:**
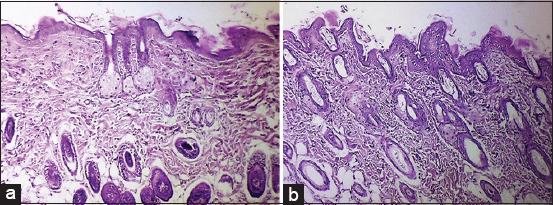
(a) Longitudinal section of normal mice skin after wound healing (CG) on day 10 showing the formation of sebaceous glands (red arrow), and the surface of the epidermis appears irregular (black arrows) with the disorder of the dermal layer (red arrow) (hematoxylin and eosin staining, 4×). (b) Longitudinal section of normal mice skin after wound healing in (CG) on day 10 showing the dermal layer, which consists of irregular collagen bundles, many fibroblasts (black arrows), and inflammatory cell infiltration (red arrow) (hematoxylin and eosin staining, 4×). CG=Control group.

In the CG, sebaceous gland formation was observed, but the epidermal surface appeared irregular, accompanied by a disorganized dermal layer. The dermis displayed irregular collagen bundles and numerous fibroblasts, along with prominent inflammatory cell infiltration, as depicted in Figure 6a and b.

**Figure 7:**
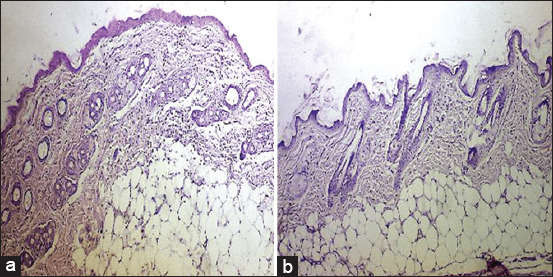
Longitudinal section of mice skin after wound healing on day 10 treated with IG-G showing, (a) new blood vessels (black arrows) with mild inflammatory cell infiltration (yellow arrows) and (b) irregular regeneration of the epidermal cells (blue arrows) and hair follicles with follicular bulbs associated sebaceous glands (red arrows) (hematoxylin and eosin staining, a and b: 4×). IG-G=Gamma radiation.

**Figure 8:**
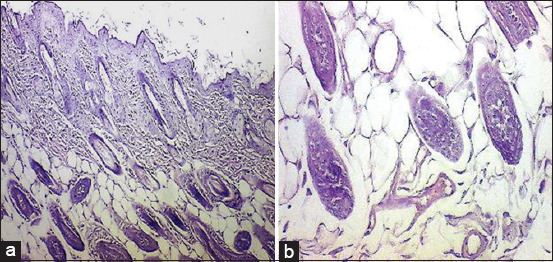
Longitudinal section of mice skin after wound healing on the 10^th^ day treated with IG-B showing, (a) hyperplasia of the epidermal cell (black arrows) with different stage formation of hair follicles (yellow arrows) and (b) new blood vessels (red arrows) and follicular bulb and papilla of a hair follicle which is the fibrovascular (blue arrows) (hematoxylin and eosin staining, (a) 4×, (b) 10×). IG-B=Beta radiation.

In IG-G, shown in Figure 7a and b, histological sections revealed newly formed blood vessels, mild inflammatory infiltration, and irregular regeneration of epidermal cells and hair follicles, including folli-cular bulbs associated with sebaceous glands. IG-B (Figure 8a and b) exhibited epidermal hyperplasia, various stages of hair follicle development, newly formed vasculature, follicular bulbs, and fibrovascular papillae.

**Figure 9:**
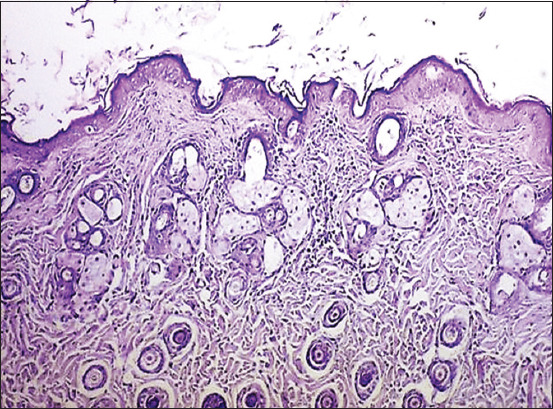
Longitudinal section of mice skin after wound healing on day 10 treated with IG-A showing hyperplasia of the epidermal cell (black arrows) with many hair follicles (red arrows) and increased collagen deposition (hematoxylin and eosin staining, 4×). IG-A=Alpha radiation.

Among all groups, IG-A demonstrated the most pronounced histological improvement, as illustrated in Figure 9. This group showed extensive epidermal hyperplasia, numerous hair follicles, and signifi-cantly increased collagen deposition. In addition, IG-A displayed fully restored dermal structures, absence of inflammatory responses, complete regeneration of the epidermis with the reappearance of a fine keratinized layer, enhanced neovascularization, orderly horizontal alignment of collagen fibers, and revitalized sebaceous glands.

## DISCUSSION

In this study, we investigated the effects of low-dose irradiation on the wound-healing process, focusing on both direct and indirect forms of ionizing radiation. This approach was motivated by the clinical challenge of impaired healing in irradiated tissues. Our findings indicate that low-dose irradiation can enhance wound healing, likely through the activation of various physiological mechanisms, including immune system stimulation [41] and bactericidal effects [42].

Ullm and Pompe [43] proposed that interactions between macrophages and fibroblasts, particularly during engagement with newly formed blood ves-sels and early granulation tissue, may strengthen the physiological processes involved in healing. Conse-quently, the density of blood vessels appears to play a crucial role in facilitating effective wound repair [44]. Supporting this, Trotsyuk *et al*. [45] demonstrated that the limited presence of blood vessels, macrophages, and neutrophils impairs healing progression in second-degree burns in rats.

Successful wound healing involves a sequential cascade encompassing hemostasis, inflammation, proliferation, and remodeling. The process begins with clot formation and transitions into an inflammatory phase [46]. Platelets release PDGF, which, in turn, activates neutrophil and macrophage recruitment and proliferation [47]. Our study is the first to report that IG-A may enhance macrophage-mediated secretion of transforming growth factor-beta, a key mechanism contributing to its superior wound-healing efficacy compared to beta and gamma radiation [48].

In addition, our findings reveal a novel role for fibroblasts, where IG-A significantly increases coll-agen synthesis, thereby improving extracellular matrix remodeling and accelerating wound closure. VEGF, secr-eted by keratinocytes, mast cells, macrophages, and fibroblasts [49], facilitates new blood vessel formation, which is essential for tissue regeneration [50].

Recent literature has shown elevated counts of fibroblasts, blood vessel segments, hair follicles, epidermal cells, and collagen deposition by day 10 post-wounding in groups treated with IG-A. Our study corroborates these findings; on day 10, IG-A showed a significantly higher number of blood vessel segments than IG-B and IG-G. In contrast, the CG exhibited the lowest vascular density at the same time point. These results are consistent with prior studies by Oh *et al*. [51] and Jabbari *et al*. [52], indicating that ionizing radiation can induce angiogenesis in both malignant and non-malignant tissues. Specifically, low-dose irrad-iation (~75 mGy) appears to promote endothelial cell migration without compromising their viability or proliferation [53].

As fibroblasts mature, they migrate and facilitate capillary formation, contributing to structural tissue support [54]. During the proliferation phase of healing, fibroblasts in granulation tissue differentiate into myofibroblasts, which are key to wound contraction [55]. They are also responsible for synthesizing collagen and other components of the connective tissue matrix, aiding in tissue regeneration and maturation [56]. In our study, fibroblast counts were significantly higher in IG-A than in the other groups, suggesting that IG-A enhances collagen synthesis and promotes a more efficient healing response. In contrast, fibroblast levels were lower in the CG on day 10, possibly compromising granulation tissue integrity. The more rapid onset of the inflammatory phase in IG-A likely triggered earlier collagen production, contributing to superior healing outcomes when compared with IG-B and IG-G. In addition, prolonged healing observed in the CG reflects delayed wound resolution.

Histological analysis revealed a reduced neutrophil presence in IG-A compared to IG-B and IG-G by day 10, suggesting that IG-A may modulate neutrophil dynamics. This modulation potentially reduces chronic inflammation and accelerates the transition to the proliferative phase. Acute inflammation is the body’s initial response to injury and plays an essential role in innate immunity. Neutrophil infiltration during early inflammation is vital for establishing tissue hemostasis [57]. Therefore, an increase in macrophage count coupled with reduced neutrophil presence, as seen in IG-A, is considered favorable for inflammation resolution [58].

In this study, mice received four 20 cGy doses of X-ray radiation administered every other day. The results suggest that such low-dose exposures may enhance innate immune responses [59]. Although much of the literature has historically emphasized the harmful effects of high-dose ionizing radiation, recent investigations suggest that exposure below specific thresholds may offer health benefits and therapeutic potential. The linear no-threshold model, which assu-mes a direct correlation between radiation dose and biological effect, has been increasingly challenged. Emerging evidence shows that low-dose ionizing radia-tion may positively influence immune modulation and disease management [60].

Our findings further support this view. On day 10, a significant increase in blood vessel density was observed at wound sites in the IG-A group compared to IG-B and IG-G. This enhancement reflects systematic advancement of granulation tissue and collagen depo-sition, along with active angiogenesis. These effects may be attributed to multiple physiological mechanisms, including improved immune responses, protection agai-nst oxidative stress through antioxidant activity, and upregulation of VEGF and PDGF, all of which contribute to neovascularization and cellular proliferation in the wound environment.

In summary, this study demonstrated significant differences between the experimental groups and the CG, with IG-A consistently outperforming IG-B and IG-G in all evaluated parameters. The maturation phase progressed more rapidly in IG-A, as confirmed by macroscopic, histological, and biochemical evidence. The accelerated healing in this group is likely attributable to the stimulatory effects of alpha irradiation, which enhanced tissue repair relative to the untreated CG.

## CONCLUSION

This study provides compelling evidence that low-dose ionizing radiation – particularly IG-A – significantly accelerates wound healing in murine models through enhanced epithelialization, increased fibroblast proliferation, collagen deposition, and neov-ascularization. Among the treatment groups, IG-A demonstrated superior efficacy, achieving complete wound closure by day 10 and yielding the highest levels of VEGF and PDGF expression, indicating potent angiogenic and regenerative responses. Beta and gamma radiation also improved healing relative to controls, but to a lesser extent.

The strength of this study lies in its integrated experimental approach, combining macroscopic wound evaluation with histological and biochemical analyses to comprehensively assess the differential effects of alpha, beta, and gamma radiation. The use of standardized injury models and controlled irradiation protocols adds to the reproducibility and translational value of the findings.

However, certain limitations must be ackno-wledged. The study was conducted in a small sample size limited to male BALB/c mice, which may not fully capture the complexity of wound healing across sexes, species, or pathological conditions such as diabetes or infection. In addition, the long-term safety and potential genomic or epigenetic effects of repeated low-dose radiation exposures were not evaluated.

Future research should explore the molecular pathways underlying radiation-induced modulation of wound healing, assess the long-term consequences of repeated exposure, and validate these findings in large animal models or clinical settings. Investigating dose optimization, radiation delivery methods, and potential synergistic effects with pharmacological agents may also broaden the therapeutic applications of ionizing radiation in regenerative medicine.

The present investigation introduces IG-A as a promising non-pharmacological modality for enhancing cutaneous tissue repair, potentially opening new aven-ues for advanced wound care strategies.

## AUTHORS’ CONTRIBUTIONS

MAK: Conceptualization, methodology, invest-igation, data analysis, and drafted, reviewed, and edited the manuscript. BMA: Growth factor concentrations analysis, methodology refinement, data interpretation, and drafted and edited the manuscript. SAHA: Inves-tigation, histological analysis, and reviewed and edited the manuscript. MNM: Analyzed the blood test data and drafted and reviewed the manuscript. All authors have read and approved the final manuscript.
